# MicroRNA-210-5p Contributes to Cognitive Impairment in Early Vascular Dementia Rat Model Through Targeting Snap25

**DOI:** 10.3389/fnmol.2018.00388

**Published:** 2018-11-13

**Authors:** Zhenxing Ren, Junlong Yu, Zimei Wu, Wenwen Si, Xianqian Li, Yuqing Liu, Jianhong Zhou, Rudong Deng, Dongfeng Chen

**Affiliations:** ^1^Department of Anatomy, The Research Center of Basic Integrative Medicine, Guangzhou University of Chinese Medicine, Guangzhou, China; ^2^College of Basic Medicine, The Research Center of Basic Integrative Medicine, Guangzhou University of Chinese Medicine, Guangzhou, China

**Keywords:** vascular dementia, miR-210-5p, Snap25, cognitive impairment, synaptic loss

## Abstract

Vascular dementia (VD) is the most common form of dementia in elderly people. However, little is understood about the role of microRNAs (miRNAs) involved in cognitive impairment in early VD. Here, a VD model induced by chronic cerebral ischemia and fetal bovine serum (FBS)-free cell model that detects synapse formation was established to investigate the function of miRNAs in early VD. The microarray analysis and real-time reverse transcription polymerase chain reaction (RT-PCR) showed that miR-210-5p increased significantly in the hippocampus of rats with 4 weeks of ischemia. The VD model rats also displayed significant cognitive deficits and synaptic loss. The overexpression of miR-210-5p decreased the synaptic number in primary hippocampal neurons, whereas specific suppression of miR-210-5p resulted in the formation of more synapses. Additionally, intracerebroventricular (ICV) injection of miR-210-5p agomir to VD rats aggravated phenotypes of cognitive impairment and synaptic loss. These VD-induced phenotypes were effectively attenuated by miR-210-5p antagomir. Moreover, bioinformatic prediction revealed that synaptosomal-associated protein of 25 KDa (Snap25) mRNA is targeted by miR-210-5p. The miR-210-5p decreased the luciferase activities of 3’ untranslated region (3’UTR) of Snap25 mRNA. Mutation of predicted miR-210-5p binding sites in the 3’ UTR of Snap25 mRNA abolished the miR-210-5p-induced decrease in luciferase activity. Western blot and immunofluorescence staining confirmed that miR-210-5p targets Snap25. Finally, RT-quantitative PCR (qPCR) and immunofluorescence staining detected that miR-210-5p agomir downregulated Snap25 expression in the cornu ammonis1 (CA1) region of hippocampi in VD rats, whereas miR-210-5p antagomir upregulated Snap25 expression. Altogether, miR-210-5p contributes to cognitive impairment in chronic ischemia-induced VD model through the regulation of Snap25 expression, which potentially provides an opportunity to develop a new therapeutic strategy for VD.

## Introduction

Vascular dementia (VD), which is characterized by a decline in learning and memory, is widely considered as the second most common cause of dementia and is expected to increase in the coming years. It presents a major public health problem. Extensive research has indicated complex mechanisms of chronic cerebral ischemia-induced VD, such as autophagy (Liu et al., 2014; Hu et al., 2017), apoptosis (Ma et al., 2017b; Aski et al., 2018; Zhao et al., 2018; Zhu et al., 2018), and Aβ and Tau mediated inflammatory response (Miao et al., 2005; Miklossy, 2008; Zhang et al., 2008), leading to neuronal death, which results in cognitive impairment and behavioral disorders. With the onset of progressive neurodegenerative disorder, VD should be focused on the early stage of pathological process, especially synaptic dysfunction. Increasing evidence has shown that lack of synaptic associated proteins (Carlson et al., 2017; Casaletto et al., 2017; Li et al., 2017a,b; Liu et al., 2017; Yao et al., 2018) contributes to synaptic dysfunction, and even slight perturbations of synapse function can lead to brain disorders (van den Maagdenberg and Plomp, 2003; Cesca et al., 2010). The mechanism of chronic ischemia-induced cognitive impairment has been studied extensively (Choi et al., 2015; Wan et al., 2017; Wang et al., 2017, 2018b; Yao et al., 2018), whereas the mechanism of synapse dysfunction in the early stage of VD remains unclear.

Synaptosomal-associated protein of 25 KDa (Snap25), which is a synapse-associated protein and a key component of soluble N-ethylmaleimide-sensitive factor attachment protein receptor (SNARE) complexes, is involved in the molecular regulation of synaptic vesicle exocytosis by linking with Ca^2+^ sensing to membrane fusion and neurotransmitter release in pre-synapse (Schiavo et al., 1997; Choi et al., 2010; Vrljic et al., 2010). However, accumulated evidence has proposed that Snap25 alteration mediates synapse plasticity as well as density, morphology and functionality of dendritic spines mainly in post-synapse (Antonucci et al., 2013; Kube et al., 2013). Moreover, transcriptional regulation of Snap25 expression has also been reported to play a great part in synaptic function (Imai et al., 2011; Furuya et al., 2012; Moravec et al., 2016; Sugeno et al., 2016).

Several microRNAs (miRNAs) have important roles in synapses: mir-34a has been found to regulate neurite outgrowth, spinal morphology and synaptic targets (Agostini et al., 2011a,b) miR-132 has been found to regulate dendritic growth and arborization of newborn neurons (Magill et al., 2010; Pathania et al., 2012) and miR-188, another synaptic active miRNA, was found to be upregulated during long-term potentiation (LTP), and it rescues the reduction in dendritic spine density (Lee et al., 2012). Our recent reports have shown that miR-125a-3p and miR-574-5p were synaptic-associated miRNAs in stroke and Alzheimer’s disease (Li et al., 2015; Liu et al., 2017). A synaptic active miRNA, miR-210, has been found to be upregulated in the serum of patients with acute cerebral infraction, which may be involved in regulating the proliferation and apoptosis of endothelial cells (Wang et al., 2018a). Some researchers have reported that overexpression of miR-210-3p disrupts the blood-brain barrier and damages dopaminergic neurons (Ma et al., 2017a), while other studies report that mir-210-3p induces neurogenesis such as sensory axon regeneration and sensory hair cell formation. (Hu et al., 2016; Riccardi et al., 2016). Moreover, the isoform, miR-210-5p, has been suggested to play a protective role in age-related molecular degeneration that may be mediated through regulating complement factor B (CFB) expression (Ghanbari et al., 2017). Also, studies on mice revealed that miR-210-5p influences the autophagic process (Frank et al., 2015). In addition, it has been reported that miR-210-5p upregulated in type 1 diabetes mellitus patients (Assmann et al., 2017) and Cushing’s disease patients. (Belaya et al., 2018). However, the correlation of miR-210 with synapse-associated proteins in the early stage of VD has not yet been studied. In the present study, we used rats after 4 weeks of cerebral ischemia, which are formed by two-vessel occlusion (2VO) to mimic the early stage of VD pathological process, and primary hippocampal neurons to establish a fetal bovine serum (FBS)-free cell model that simulates synapse dysfunction in early VD, to further explore the functions of miRNAs in synaptic loss that are involved in cognitive impairment.

## Materials and Methods

### Animals and Materials

All experiments were approved by the Experimental Ethics Committee of Guangzhou University of Chinese Medicine. A total of 60 male Sprague–Dawley (SD) rats averaging 180–220 g in weight were provided by Guangzhou University of Chinese Medicine (SCXK (Yue) 2013-0034). Rats were maintained at a constant temperature of 25 ± 0.1°C on a 12-h light/dark cycle (lights on at 07:30) with *ad libitum* access to food and water. They were group-housed with five rats per cage and were kept adaptive for breeding for at least 1 week prior to the surgery.

### Experiment Protocol

#### Experiment 1

To observe the cognitive function and synaptic loss in VD model rats, rats which had 4 weeks of chronic ischemia were prepared through the 2VO model. Animals were randomly distributed into two groups: the control group and the VD model group.

#### Experiment 2

To validate how miR-210-5p agomir suppresses hippocampal Snap25 expression, animals were randomly divided into four groups. The groups were control, VD model, agomir (2VO + miR-210-5p agomir) and antagomir group (2VO + miR-210-5p antagomir). When the behavior test was completed, the rats (*n* = 13/group) were anesthetized and sacrificed, with fresh brains divided from central sulcus into two parts, one was quickly removed to −80°C (*n* = 13/group), while the other was fixed using 4% PFA (*n* = 6/group) and 2.5% glutaraldehyde (*n* = 6/group).

### Permanent Common Carotid Artery Occlusion

The rats used in the experiments were anesthetized by an intraperitoneal (i.p) injection using 3.5% chloral hydrate (300 mg/kg). In the VD model rats, the bilateral common carotid arteries were carefully exposed through a midline incision and were permanently double-ligated with silk sutures. In the control group, the same procedures were performed except for the occlusion of the bilateral common carotid arteries.

### Intracerebroventricular Injection of Mimic and Inhibitor

After 4 weeks of chronic ischemia, rats in each group were subjected to 3 days of continuous intracerebroventricular (ICV) injection separately. The miR-210-5p agomir (50 pmol/rat) and antagomir (100 pmol/rat) were dissolved in deionized water immediately before use and injected with a volume of 2.5 μl into each lateral ventricle (miR-210-5p agomir and antagomir were purchased from RiboBio Co., Ltd). The rats of the control group and VD model group were injected with the same volume of normal saline. When anesthetized with 3.5% chloral hydrate (300 mg/kg; i.p), the rats were mounted on the stereotaxic instrument, with its back on the panel. In short, two small holes must be carefully drilled into the skull bilaterally, using a surgical drill according to the rat brain atlas in order to deliver the agomir and antagomir solutions into the lateral ventricle. The stereotaxic coordinates for ICV injection were as follows: −0.9 mm anterior, 1.8 mm lateral and −3.8 mm from the bregma. The details about ICV injection were shown in Supplementary Material. Penicillin G (80,000 units/rat, diluted in 0.9% sodium chloride, i.p) was used to avoid infection during surgery and ICV injection. The rats were anesthetized before ICV injection.

### Morris Water Maze

Spatial learning and memory were tested after 24 h of the last ICV injection by a modification of the procedure described by Morris. A circular pool (150 cm in diameter, 60 cm in height) was filled to a depth of 30 cm with water at a temperature of 22 ± 1°C. The pool was divided into four quadrants of equal area. These quadrants were NE, NW, SE and SW. A platform (20 cm in diameter) was placed 1 cm below the pool surface, which is midway between the center and rim of the pool in the NE quadrant throughout the entire experiment. On day 1, the rat was placed into the pool in the SW quadrant and the time taken by the rat to find the escape platform was recorded. If it failed to do so within 200 s, it was placed on the platform for 15 s and removed from the pool. From day 2, the rat was given four trials a day for five consecutive days between 9:00 and 17:00 with an intertrial interval of 15 min, which was subjected to the acquisition of spatial learning. Later, the probe trials were run in the same way as the four trials on day 6, during which the platform was removed from the pool with the rats that were capable of swimming freely to detect the memory. After each trial, the rats were taken out, dried, and put into a separate cage.

### MicroRNA Microarray Assay

The hippocampi were taken to miRNA microarray assay, which were performed at Guangzhou RiboBio Co., Ltd. The experiment that was performed included pre-hybridization, hybridization, washing and imaging. GustomArray™ microarray was assembled with hybridization cap and clips. The microarray was rinsed to decrease specific hybridization background, was covered with the imaging solution, and was loaded into the GenePix 400 B microarray Scanner to scan.

### Primary Hippocampal Neuron Culture and Transfection

To acquire primary hippocampal neuron, the pregnant rat at E17–E19 days of gestation was used as the material. The pregnant rat was sacrificed by cervical dislocation and opened at the abdomen to remove the two horns of the uterus with sterile scissors. The pups were taken out with placental sacs and broken away from umbilical cord, and then, their heads were cut off. The brain was exposed on the stage of the stereo microscope with forceps peeling away the scalp and the skull. The hippocampus would be found when the cortices were taken off following the willis circle and were detached clearly from the brain without any existed meninges. Later, the hippocampus was washed with pre-cooled phosphate buffered saline (PBS) that contained 1% penicillin-streptomycin and was moved into 6 ml DMEM/F12 medium (Gibico) that contained papain (2 mg/ ml, solabro) for digestion. It was digested in a 37°C incubator for 20 min with a gentle shake every 5 min. A total of 1 ml FBS (Gibico) was added in to terminate digestion. Cell suspension was gained through 200-mesh sieve. The digestion was repeated three times. The collections were centrifuged at 1,000 rpm for 8 min and then suspended in the culture medium containing 10% FBS. We had calculated the viable cell rate up to 86% by using the trypan blue staining. As per recommendation, 7 × 10^5^ viable cells were plated with 35 mm Bottom Petri Dish (φ = 20 mm, Nest) for immunofluorescence staining and 7 × 10^5^ viable cells per well in six cluster plates for quantitative PCR (qPCR) and western blot analysis. Cells were divided into four groups: FBS group (cultured with 10% FBS added medium), FBS-free group (cultured with FBS-free medium), miR-210-5p mimic group (cultured with FBS-free medium and transfected with miR-210-5p mimic) and miR-210-5p inhibitor group (cultured with FBS-free medium and transfected with miR-210-5p inhibitor), and were maintained at 37°C with humid 5% CO_2_/95% air condition (miR-210-5p mimic and inhibitor were purchased from RiboBio Co., Ltd). The medium was changed to a neurobasal medium (with 2% B27) after 24 h and then half-changed at the interval of 3 days. The neurites appeared clearly around 8–10 days.

### PC12 Cell Culture and Transfection

To certify that miR-210-5p targets Snap25 mRNA 3’ untranslated region (3’UTR), PC12 cells were cultured in 1640 RPMI medium (Gibico) supplemented with 10% FBS and 1% mixture liquid of penicillin/streptomycin and were incubated at 37°C in a humid 5% CO_2_/95% air environment. The PC12 cells were plated with 24-well cluster plates at a density of 2 × 10^4^ cells per cm^2^. As a control group, PC12 cells were transfected using Lipofectamin 2000 with psiCHECK2 vector, and the others were co-transfected using plasmids and mimic or inhibitor (200 ng psiCHECK2-Snap25 wild-type (WT) plasmid, 200 ng psiCHECK2-Snap25 Mut plasmid, 100 nM miR-210-5p mimic and 200 nM miR-210 inhibitor).

### Western Blot Analysis

The protein was extracted from the frozen fresh hippocampus tissue by RIPA lysis buffer (Thermo) with 1% phenylmethanesulfonyl fluoride (PMSF). The supernatant of each group was obtained by centrifugation of 12,000 g at 4°C for 20 min and quantified with bicinchoninic acid (BCA) protein assay (bioWORLD). Lysates were separated by 10% sodium dodecyl sulfate polyacrylamide gel electrophoresis (SDS-PAGE) and blotted onto polyvinylidene difluoride (PVDF) membranes (0.45 μm, Millipore, Germany). Then, 5% bovine serum albumin (BSA) dissolved in tris buffered saline with Tween (TBST) buffer containing 0.2% Tween 20 was used as blocking buffer. The primary antibodies were rabbit anti-Snap25 (1:500; 14903-1-AP, Proteintech) and rabbit anti-glyceraldehyde-3-phosphate dehydrogenase (GAPDH; 1:10,000; ab181602, abcam). The secondary horseradish peroxidase (HRP)-conjugated goat anti-rabbit antibody (1:20,000; ab6721, abcam) was visualized by enhanced chemiluminescence with a western blot detection kit (Millipore), according to the manufacturer’s instructions and analyzed by using imageJ software (USA). The signal of Snap25 was normalized to the housekeeping protein GAPDH and detected in the same blot, and then ratios with average control values were determined.

#### Real-Time Quantity PCR Analysis

The total RNA lysates were prepared using Trizol Reagent (Life, 139505) for 30 min on ice and then isolated using Direct-zol™ RNA MiniPrep Plus (Zymo Research, ZRC200606). The quantity and the quality of RNAs were analyzed on a NanoDrop 1,000 spectrophotometer (Thermo Fisher Scientific). Snap25 mRNA and miR-210-5p were done on cDNAs that were prepared using Transcriptor First Strand cDNA Synthesis Kit (Roach), with a mix of dT primers for mRNA and stem loop primers (Sangong, Shanghai) for miRNA, at 50°C for 1 h. The qPCR was performed using Faststart Universal SYBR Green (Roach) with appropriate primers. Relative quantification of gene expression was conducted with the Bio-Rad CFX96 (Bio-Rad) and Real-Time PCR System. Relative quantification was carried out with the 2^−ΔΔCt^ method. The GAPDH mRNA was used as an internal control for mRNA expression and U6 for miRNA. The primers are shown as follows:

- miR-210-5p RT: 5’ GTCGTATCCAGTGCAGGGTCCGAGGTATTCGCACTGGATACGACCAGTGT 3’
- miR-210-5p Forward: 5’ CATGAGCCACTGCCCACAGC 3’
- miR-210-5p Reverse: 5’ ATCCAGTGCAGGGTCCGAGG 3’
- U6 RT: 5’ GTCGTATCCAGTGCAGGGTCCGAGGTATTCGCACTGGATACGACAAAATA 3’
- U6 Forward: 5’ AGAGAAGATTAGCATGGCCCCTG 3’
- U6 Reverse: 5’ ATCCAGTGCAGGGTCCGAGG 3’
- Snap25 Forward: 5’ CATGGGCAATGAGATTGACA 3’
- Snap25 Reverse: 5’ CCACTTCCCAGCATCTTTGT 3’
- Gapdh Forward: 5’ GGTGGACCTCATGGCCTACA 3’
- Gapdh Reverse: 5’ CTCTCTTGCTCTCAGTATCCTTGCT 3’

#### Immunofluorescence Staining

To observe the effect of miR-210-5p on Snap25 protein expression, primary hippocampus neurons that were cultured for 8 days were transfected with 100 nM miR-210-5p mimic or 200 nM miR-210-5p inhibitor for 6 h according to the protocol of Lipofectamin 2000 and were terminated by using fresh neurobasal medium containing 2% B27. As a control group, primary hippocampus neurons were treated with the same procedure using only Lipofectamine 2000 for 6 h and were terminated in the same way. After 24 h of transfecting termination, cells were fixed with 4% PFA for 30 min at room temperature, permeabilized with 0.1% Triton X-100/PBS for 10 min subsequently, and then processed incubation with primary antibody Snap25 overnight at 4°C. On the second day, PBS washes were performed three times, after which secondary antibody Alex-647 coupled Goat anti rabbit were incubated for 1 h at room temperature. And then, Phalloidin (1:1,000; ab, abcam) coupled with Alex-488 (1:1,000; ab, abcam) were incubated for 1 h at room temperature, and nuclei were visualized by staining with diamidino-2-phenyl-indole-dihydrochloride (DAPI; Solabo).

To further investigate whether miR-210-5p reduced expression of Snap25 protein would affect synaptic density, primary hippocampus neurons were formed as before. After 6 h transfection, the FBS-free groups were changed to neurobasal medium containing 2% B27, except for the FBS group, in which the medium was changed with additional 10% FBS. Later, the staining process was performed as usual. The primary antibodies were Snap25 (1:250, 14903-1-AP, Proteintech) Synapsin 1 (Syn1; 1:1,000; ab8, abcam) and postsynaptic density protein 95 (PSD95; 1:1,000; ab13552, abcam), and the secondary antibodies were Alex 488 (1:1,000; ab150077, abcam) and Alex 647 (1:1,000; ab150179, abcam).

The hippocampus, which was fixed at 4% PFA for 48 h and 30% (w/v) sucrose for 48 h, was made into frozen sections, permeabilized with 0.5% Triton X-100/PBS for 30 min at room temperature, and the subsequent operating procedure was the same as the PC12 cells. The cornu ammonis1 (CA1) region of the hippocampus was focused and visualized through confocal microscope (ZESS).

#### Transmission Electron Microscope

A transmission electron microscope (TEM) was used to visualize synapses in CA1 area of the hippocampus. The brain tissues were fixed with 2.5% (w/v) glutaraldehyde that was diluted in cacodylate buffer (0.1 M, pH 7.4) for 1 h at 4°C, incubated in Tris–HCl (0.05 M, pH 9.0) containing diaminobenzidine (2.5 mg/ml) and H_2_O_2_ (10 μl/ml of a 3% solution) for 1 h at 21°C, washed in cacodylate buffer (0.1 M, pH 7.4) for 5 min at 21°C, post-fixed in 1% (w/v) osmium tetroxide that was diluted in cacodylate sodium solution (0.1 M, pH 7.4) for 1 h at 21°C in the dark, and rinsed in cacodylate buffer (0.1 M, pH 7.4). All incubation was processed in the absence of light. The preparations were then dehydrated in graded ethanol solutions and embedded in epon. Ultrathin sections were cut with an ultramicrotome, contrasted with uranyl acetate and lead citrate, and detected under TEM (JEOL 2100F, Japan).

#### DNA Constructs and Luciferase Reporter Assay

The complete Snap25 mRNA 3’ UTR containing miR-210-5p sites was amplified from rats’ genomic DNA and cloned into XhoI and NotI sites of psiCHECK2 vector (Promega), generating psiCHECK2-Snap25 WT plasmid. The point mutations of miR-210-5p seed binding sites were produced by overlapping PCRs into Snap25 3’ UTR plasmid using synthetic oligonucleotides, resulting in psiCHECK2-Snap25 Mut plasmid (CAGUGGC to GUCACCG; Figure 5A). All constructs were verified by sequencing. In the plasmid, the inserted fragment was placed after the Renilla luciferase (hRluc) region, and relatively, firefly luciferase served as an internal reference (Figure 5B upper).

**Figure 1:**
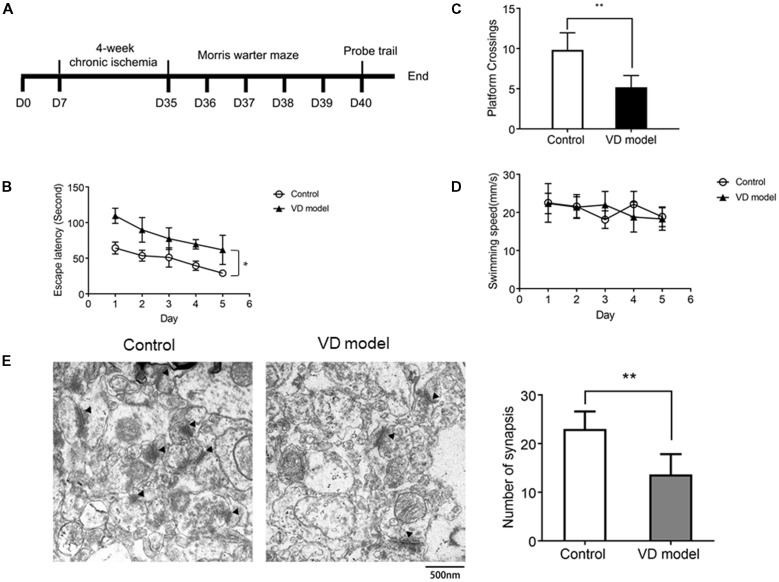
Chronic ischemic rat model exhibits the decline of spatial learning capability (*n* = 13/group) and synapse loss in the hippocampus relative to control group (*n* = 6/group). **(A)** The timeline of animal experiment. **(B)** The escape latency of individual rats was processed by a Morris water maze that lasts for 5 days (four times per day). **(C)** The number of platform crossings measured within 120 s. **(D)** The motor function was assessed by the swimming speed. **(E)** Synapses were indicated with black triangles; bar chart showed the number of synapses. Data are shown as mean ± standard deviation (SD) values, and statistical significance between both groups is defined as *p < 0.05; **p < 0.01.

**Figure 2:**
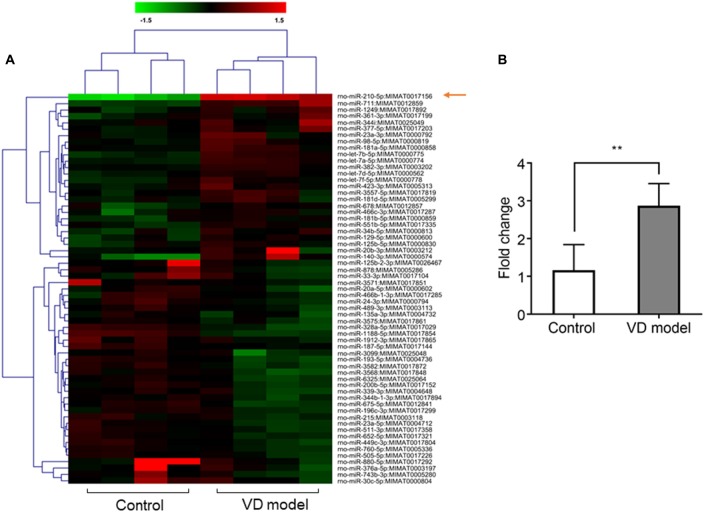
The expression of microRNA (miRNA)-210-5p is significantly increased in hippocampal tissues of vascular dementia (VD) model rats when compared with control rats. **(A)** miRNA profile was measured through microarrary (*n* = 4/group). In cluster diagram, red color represents relatively high quantity of the miRNAs expression, and the green means relatively low. **(B)** mir210 expression was tested by quantitative PCR (qPCR; *n* = 6/group). Data are shown as mean ± SD values, and statistical significance between both groups is defined as **p < 0.01.

**Figure 3:**
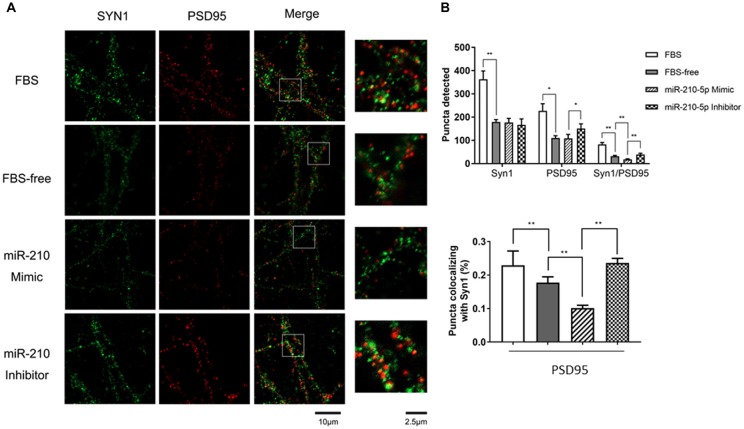
miR-210-5p downregulates postsynaptic density. (*n* = 3/group, 2 images per sample). **(A)** Green puncta were stained using Synapsin1 (Syn1) antibody, which represents pre-synapses. Red puncta stained with PSD-95 means post-synapses. The puncta colocalized with Syn1 and PSD-95 indicates functional synapses. **(B)** Quantification results of puncta in each channel; the colocalized synaptic density is normalized by pre-synapses respectively. Data are shown as mean ± SD values, and statistical significance between both groups is defined as *p < 0.05; **p < 0.01.

**Figure 4:**
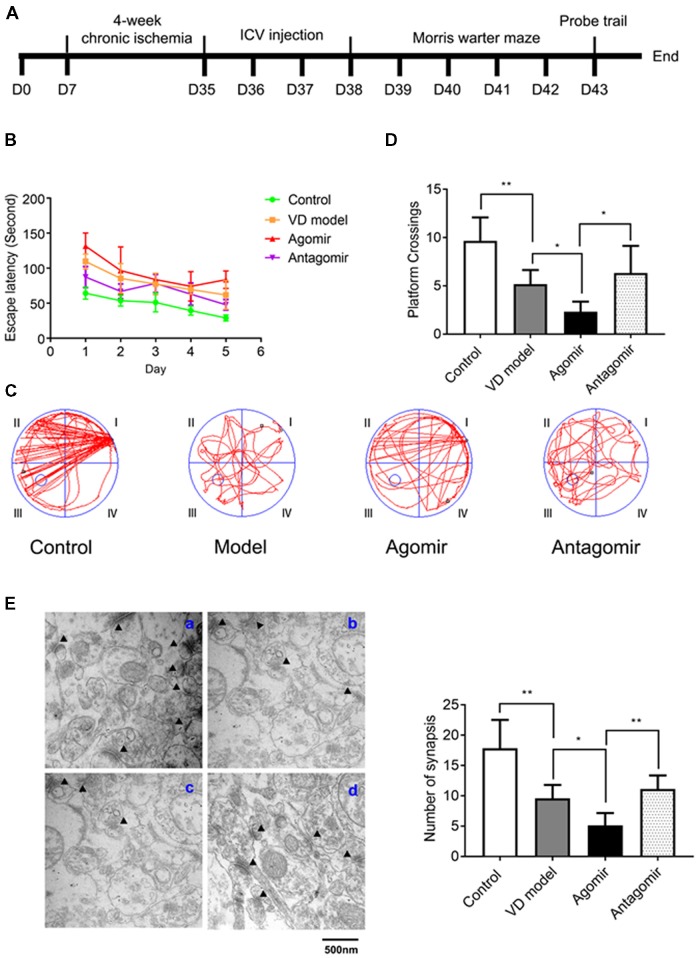
Intracerebroventricular (ICV) injection of miRNA-210-5p agomir aggregates cognitive impairment level and antagomir attenuates cognitive impairment in VD model (*n* = 13/group). **(A)** Timeline. **(B–D)** The latency, the moving trails of rats in platform crossing test and number of rats crossing over the platform. **(E)** Synapses were indicated with black triangles; a: Control group, b: Model group, c: Agomir group, d: Antagomir group; bar chart showed the number of synapses (*n* = 6/group). Data are shown as mean ± SD values, and statistical significance between both groups is defined as *p < 0.05; **p < 0.01.

**Figure 5:**
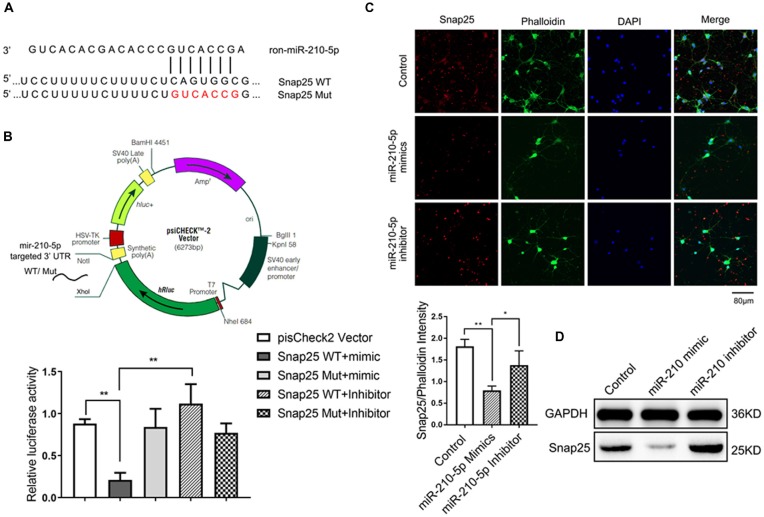
miR-210 can specifically target on synaptosomal-associated protein of 25KDa (Snap25; *n* = 3/group, 2 images per sample). **(A)** The target sites of miR-210-5p in Snap25 mRNA 3’ untranslated region (3’UTR) were analyzed by Targetscan 7.0. **(B)** The construction profile of psiCHECK2-Snap25 wild-type (WT) and Mut vectors are shown above. Snap25 luciferase reporter (Snap25 3’ UTR WT) and the corresponding mutant construct (Snap25 3’ UTR Mut) were co-transfected into PC12 cells together with miR-210 mimics and inhibitor respectively. Luciferase activity was measured 24 h post transfection. Values are expressed relatively to the internal firefly luciferase activity. **(C)** Immunofluorescence and its intensity analysis showed miR-210-5p mimics decreased Snap25 protein expression in Primary neuron cells, whereas miR-210-5p inhibitor increased snap 25 protein expression. **(D)** Western blot results showed a decrease of Snap25 protein expression in primary neuron cells treated with miR-210-5p mimics, whereas they showed an increase of snap 25 expression in cells with miR-210-5p inhibitor. Data are shown as mean ± SD values, and statistical significance between both groups is defined as *P < 0.05; **p < 0.01.

### Statistics

All data in our study were expressed as mean ± standard deviation (SD). Analysis of variance (ANOVA) was carried out with Graphpad Prism7 (USA). Statistical comparison between two groups was performed by Student’s *t*-test. A two-way ANOVA test was applied for escape latency analysis and the swimming speed test. A one-way ANOVA test was used to analyze other variances for comparison of multiple groups. Difference was considered statistically significant at *p* < 0.05. The fold-changes of qRT-PCR were considered statistically significant at fold-change ≥2.0 or fold-change ≤0.5 and *p* < 0.05.

## Results

### Chronic Ischemic Rats Exhibit Impaired Acquisition of Spatial Learning and Synapse Loss

The morris water maze test was applied to assess the early symptoms of dementia in rats that are subjected to 4 of weeks ischemia (Figure 1A). When compared with control model rats, VD model rats were unable to locate the submerged platform, although there were related clues as a position reference; these model rats observed longer latency and slower improvements (Figure 1B). In the probe trials, VD rats used less time to cross over the platform (Figure 1C). These behaviors indicate cognitive impairment in rats with chronic ischemia. Moreover, there was no difference in swimming speed between VD rats and control rats (Figure 1D), ensuring that abnormal behavior of VD rats is unrelated to motor impairment. Further, by using TEM, we observed that the number of synapses was significantly reduced in the hippocampus of VD rats (Figure 1E). These findings reveal cognitive impairment and hippocampal synaptic loss in VD model rats.

### The miR-210-5p Expression Is Increased in Hippocampus of VD Rats

Based on microarray analysis, the expression of miRNAs was profiled in the hippocampus of VD models and control rats (*n* = 4/group). Using the quantile normalization method, an average was taken of the repeated data from the same sample. The *P*-value was calculated with Rank product method. A total of 61 miRNAs were statistically changed between both groups with 30 miRNAs being upregulated, while 41miRNAs were downregulated (Figure 2A). Among them, miR-210-5p was upregulated most significantly in the hippocampus of VD rats. Furthermore, RT-qPCR detection also showed a 2.5-fold increase in the expression of hippocampal miR-210-5p in VD rats (Figure 2B). This increase suggests a strong association of increased miR-210-5p levels with chronic ischemia-induced VD.

### Overexpression of miR-210-5p Reduces Synapse Density

To investigate whether increased miR-210-5p levels would give rise to synaptic loss, we performed immunofluorescence to detect the synapse density. The FBS-free medium cultured neurons showed less pre-synapses and post-synapses with respect to those that were processed with 10% FBS-contained medium. Additionally, the primary neurons transfected with miR-210-5p mimic showed less post-synapses and lower density of colocalized synapses when compared with the FBS-free group. The miR-210-5p inhibitor could increase the postsynaptic density and colocalized synapses (Figure 3A).

Interestingly, the FBS-free induced groups showed no significance between each other. Moreover, post-synaptic density and colocalized synaptic density showed significant reduction in FBS-free induced groups. Additionally, the miR-210-5p mimic further decreased the colocalized synaptic density when compared with FBS-free group. On the contrary, miR-210-5p inhibitor increased the colocalized synaptic density (Figure 3B, upper). Subsequently, we analyzed the colocalized synapses by normalizing with pre-synapse (Syn1), which indicated that FBS-free processing will give birth to lower normalized post-synapses, which is 17.76%, when compared with the FBS group, which is 22.93%. Importantly, miR-210-5p significantly downregulated the normalized postsynaptic density to 10.16%, while miR-210-5p inhibitor reversely upregulated normalized postsynaptic density to 23.65%. (Figure 3B, down).

### miR-210-5p Is Involved in Cognitive Impairment of VD Model Rats

To assess whether miR-210-5p could influence VD model rats’ spatial learning and memory, we performed 2 VO surgeries on rats again (Figure 4A). Rats that were operated but did not undergo VO were made as the control group. By the Morris water maze testing, miR-210-5p agomir aggravated the latency of rats with VD, while the antagomir can attenuate the latency of rats with VD (Figure 4B). Representative swimming path during the trial probe (Figure 4C) has shown that crossing over the platform of VD model rats decreased significantly when compared with the control group rats (*n* = 13/group). It has also been shown that VD model rats that were treated with miR-210-5p agomir showed less crossing than the model rats, while miR-210-5p antagomir increased the number of rats crossing over the platform (Figure 4D). Later, we performed TEM detection to investigate hippocampal synapses in each group of rats. The number of synapses illustrated in the VD model group rats’ hippocampus declined dramatically when compared with control group rats; miR-210-5p agomir reduced the synapses further as to model rats; however, miR-210-5p antagomiR-treated model rats showed an increased number of synapses (Figure 4E).

### miR-210-5p Specifically Targets on Snap25

To determine if the increased miR-210-5p leads to synaptic loss in the hippocampus, we analyzed its potential targets using a bioinformatic program. The result predicted that miR-210-5p may target Snap25 3’ UTR binding sites (binding sites are shown in Figure 5A).

To clarify whether miR-210-5p directly binds to the 3’ UTR of Snap25, we investigated it in PC12 cells. Dual-luciferase reporter plasmids (psiCHECK2 Vector) carrying the Snap25 3’ UTR with WT or base-pair mutant miR-210-5p binding regions were constructed (Figure 5B, upper). After co-transfection of PC12 with psi-CHCEK2-Snap 3’ UTR and miR-210-5p mimic and inhibitor respectively, the luciferase activity decreased markedly when co-transfected with psi-CHCEK2-Snap WT and miR-210-5p mimic in comparison with empty vector, psi-CHCEK2-Snap WT, psi-CHCEK2-Snap Mut and miR-210-5p mimic or inhibitor (Figure 5B, down).

In addition, immunofluorescence staining and western blot provided the evidence that Snap25 expression decreased through the infection of primary hippocampal neurons with miR-210-5p mimic when compared with the control group, whereas miR-210-5p inhibitor increased the expression of Snap25 (Figures 5C,D). These findings suggest that miR-210-5p regulates Snap25 by direct interaction with its 3’ UTR.

### miR-210-5p Agomir Reduces Snap25 Expression in Hippocampus of VD Model Rats

**Figure 6:**
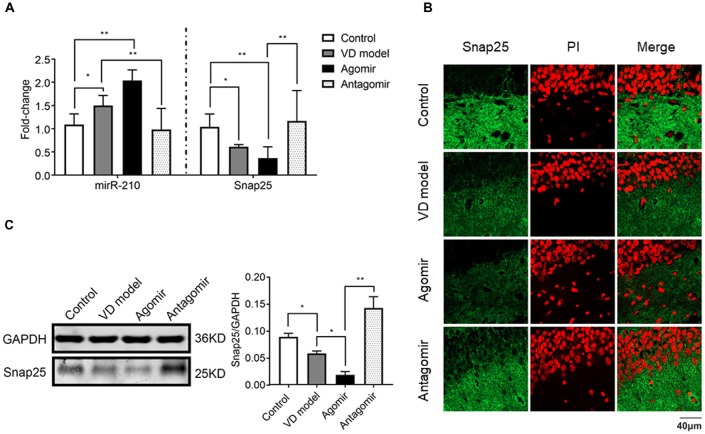
ICV injection of miRNA-210 antagomir effectively restore hippocampal snap25 expression in VD model (*n* = 6/group). **(A)** miR-210-5p and Snap25 mRNA expression detected by qPCR (*n* = 6/group). **(B)** Snap25 protein expression visualized through immunofluorescence. **(C)** Snap 25 protein expression in hippocampus tested by western blot (*n* = 6/group). Data are shown as mean ± SD values, and statistical significance between both groups is defined as *p < 0.05; **p < 0.01.

To further find out whether miR-210-5p regulates Snap25 expression *in vivo*, we detected the Snap25 mRNA level using RT-qPCR (*n* = 6/group), immediately after a probe trial of the Morris water maze, obtained by agomir and antagomir ICV injected into the hippocampus, as well as the equal volumes of normal saline for control and model group rats. A significant increase of miR-210-5p expression and reduction of Snap25 mRNA level was observed in the hippocampus of VD model rats when compared with control group rats (Figure 6A). With miR-210-5p agomir ICV injected in the hippocampus, miR-210-5p levels further increased and the Snap25 level reduced in comparison with the model group (Figure 6A). However, miR-210-5p antagomir downregulated miR-210-5p, whereas they upregulated Snap25 mRNA level in hippocampus with respect to those rats treated with agomir (Figure 6A). Results from immunofluorescence staining and western blot confirmed that the expression of Snap25 protein was significantly reduced in the hippocampus of VD rats when compared to control group rats. The miR-210-5p agomir can result in downregulation of Snap25 expression *in vivo*, whereas antagomir increased Snap25 protein expression in the hippocampus of VD rats (Figures 6B,C).

## Discussion

In the present study, we investigated the roles played by miRNAs in synaptic loss, involving spatial learning and memory. The evidence demonstrated that miR-210-5p mediates the cognitive impairment effects of chronic cerebral ischemia-induced VD by suppressing the expression of Snap25, which leads to synaptic loss. The major findings of the present study were as follows: (1) Rats with 4 weeks of chronic ischemia displayed a significant synaptic loss and cognitive deficits; (2) miR-210-5p was increased significantly in the hippocampus of VD model rats and Snap25 mRNA is targeted by miR-210-5p; and (3) miR-210-5p agomir aggregates cognitive impairment of VD model and leads to more synaptic loss, and miR-210-5p agomir downregulated Snap25 mRNA level and protein expression *in vivo*. These findings suggest that the dysfunction of the miR-210-5p–Snap25 signaling pathway induced by chronic ischemia might be a causative event leading to the synaptic loss in VD.

One of the major findings of this study shows that synaptic loss in the early stage of VD is linked to cognitive impairment. Animal models have helped us understand how chronic ischemia contributes to VD. Chronic cerebral hypoperfusion precedes the onset of VD (Román et al., 1993) and it may be a trigger for VD (Kasparova et al., 2005). The permanent occlusion of the bilateral (left and right) common carotid arteries (2VO model) was a good way to study VD in rodent models, which has similarity to the VD development process (Farkas et al., 2004). Studies have evaluated synaptic change, which indicates that synapse loss is probably not a hallmark specific to Alzheimer’s disease but rather a change common to many diseases associated with dementia (Scheff et al., 2014). In this study, we found that the rats displayed significant cognitive deficits after 4 weeks of chronic ischemia, suggesting that synaptic pathology in the early stage of VD is linked to cognitive impairment. Our findings were consistent with Yi Shu’s work (Shu et al., 2013). We focused on the early stage of VD, where the rats with 4 weeks of chronic ischemia were proved to be available. This allowed the identification of miRNAs that are regulated in response to ischemic insult from loss of synapses. We have found and confirmed numerous upregulated or downregulated miRNAs by screening through a microarray, among which miR-210-5p appears most significantly overexpressed in the hippocampus of VD model rats.

The miRNA-mediated inhibition of translation is an attractive mechanism that can precisely control gene expression in neurons. miR-153 has been reported to regulate synaptic transmission and neuronal development (Wei et al., 2013). miR-199a-5p shows protection of the spinal cord via downregulation of endothelin-converting enzyme-1 (ECE1) in rats (Bao et al., 2018). miR-134 is reported to regulate synaptic plasticity. Additionally, dendritic localized pre-miR-134 promotes hippocampal neuron dendritogenesis (Zampa et al., 2018). miR-210-5p is a member of miRNAs that are involved in the regulation of neuronal function. Differently, our data indicates that overexpression of miR-210-5p decreases synapse density. miR-210-5p also likely targets other mRNAs, but miR-210-5p–Snap25-postsynapse pathway is sufficient to explain the role of miR-210-5p regulation of synaptic loss and cognitive impairment in early VD.

Previous studies have shown that Snap25 is a key component of the SNARE complex, which mediates vesicle fusion (Horváth et al., 2017; Lou et al., 2017; Shi et al., 2017; Zurawski et al., 2017). Extensive SNAREs have been reported to play functional roles of synaptic vesicles docking to presynaptic membrane in neurons (Batista et al., 2017; Van Hook et al., 2017). Snap25 provides two α-helix domains, which contributes to SNARE proteins assembling into trans-SNARE complexes to provide the force that is necessary for vesicle fusion (Yang et al., 2000). Unexpectedly, reduction of Snap25 not only impairs vesicle release, but also increases neurotransmission (Antonucci et al., 2013). However, the absence of Snap25 leads to the pool of vesicles tending to be empty and the abolishment of fast calcium-triggered exocytosis (Sørensen et al., 2003). Moreover, Sandra Jurado’s (Jurado et al., 2013) work supports that the deficit of LTP induction is caused by a decrease in NMDAR-mediated synaptic currents. Additionally, in the brain, Snap25 is a part of a molecular complex including PSD95 and p140Cap, which impacts on the spine number of CA1 hippocampal region in mice (Kube et al., 2013; Fossati et al., 2015). These findings suggest that in postsynapse, reduction of Snap25 causes postsynaptic loss as well as learning and memory. In the current study, Snap25 was directly downregulated by miR-210-5p *in vitro* and *in vivo*, which confirms that overexpression of miR-210-5p leads to cognitive defects.

In summary, our data demonstrate that miR-210-5p contributes to cognitive impairment in early VD in rats though targeting Snap25. Precise control of Snap25 levels by miR-210-5p can be a potential medical benefit for Snap25 associated diseases such as attention deficit hyperactivity disorder, schizophrenia, early-onset of bipolar disorders and so on. Finally, improving our understanding about the molecular mechanisms of miR-210-5p that contribute to protective synapses may be an interesting point for novel therapeutic intervention in VD.

## Author Contributions

RZ contributed to the design of the work and drafting the article. JY and WS worked on the experiments such as animal associated experiments and molecular biology. ZW and XL aided culture cells and performed cell experiments. YL performed the data collection and analysis. JZ and RD provided technical assistance. DC provided the conceptions and lot of guidance for this project and made the final approval of the version to be published.

## Conflict of Interest Statement

The authors declare that the research was conducted in the absence of any commercial or financial relationships that could be construed as a potential conflict of interest.

## Abbreviations

2VO: two vessel occlusions
CA1: cornu ammonis1
i.p: intraperitoneal injection
ICV: intracerebroventricular
miRNAs: microRNAs
PSD95: postsynaptic density protein 95
Snap25: Synaptosomal-associated protein of 25 KDa
Syn1: synapsin1
VD: vascular dementia.

## References

[B1] Agostini M. Tucci P. Killick R. Candi E. Sayan B. S. Rivetti di Val Cervo P.. (2011a). Neuronal differentiation by TAp73 is mediated by microRNA-34a regulation of synaptic protein targets. Proc. Natl. Acad. Sci. U S A 108, 21093–21098. 10.1073/pnas.1112061109 22160687 PMC3248477

[B2] Agostini M. Tucci P. Steinert J. R. Shalom-Feuerstein R. Rouleau M. Aberdam D.. (2011b). microRNA-34a regulates neurite outgrowth, spinal morphology and function. Proc. Natl. Acad. Sci. U S A 108, 21099–21104. 10.1073/pnas.1112063108 22160706 PMC3248521

[B3] Antonucci F. Corradini I. Morini R. Fossati G. Menna E. Pozzi D.. (2013). Reduced SNAP-25 alters short-term plasticity at developing glutamatergic synapses. EMBO Rep. 14, 645–651. 10.1038/embor.2013.75 23732542 PMC3701242

[B4] Aski M. L. Rezvani M. E. Khaksari M. Hafizi Z. Pirmoradi Z. Niknazar S.. (2018). Neuroprotective effect of berberine chloride on cognitive impairment and hippocampal damage in experimental model of vascular dementia. Iran. J. Basic Med. Sci. 21, 53–58. 10.22038/IJBMS.2017.23195.5865 29372037 PMC5776437

[B5] Assmann T. S. Recamonde-Mendoza M. De Souza B. M. Crispim D. (2017). MicroRNA expression profiles and type 1 diabetes mellitus: systematic review and bioinformatic analysis. Endocr. Connect. 6, 773–790. 10.1530/ec-17-0248 28986402 PMC5682418

[B6] Bao N. Fang B. Lv H. Jiang Y. Chen F. Wang Z.. (2018). Upregulation of miR-199a-5p protects spinal cord against ischemia/reperfusion-induced injury via downregulation of ECE1 in rat. Cell. Mol. Neurobiol. 38, 1293–1303. 10.1007/s10571-018-0597-2 29948551 PMC11481941

[B7] Batista A. F. R. Martinez J. C. Hengst U. (2017). Intra-axonal synthesis of SNAP25 is required for the formation of presynaptic terminals. Cell Rep. 20, 3085–3098. 10.1016/j.celrep.2017.08.097 28954226 PMC5659736

[B8] Belaya Z. E. Grebennikova T. A. Melnichenko G. A. Nikitin A. G. Solodovnikov A. G. Brovkina O. I.. (2018). Effects of endogenous hypercortisolism on bone mRNA and microRNA expression in humans. Osteoporos. int. 29, 211–221. 10.1007/s00198-017-4241-7 28980049

[B9] Carlson S. W. Henchir J. Dixon C. E. (2017). Lateral fluid percussion injury impairs hippocampal synaptic soluble N-ethylmaleimide sensitive factor attachment protein receptor complex formation. Front. Neurol. 8:532. 10.3389/fneur.2017.00532 29067000 PMC5641299

[B10] Casaletto K. B. Elahi F. M. Bettcher B. M. Neuhaus J. Bendlin B. B. Asthana S.. (2017). Neurogranin, a synaptic protein, is associated with memory independent of Alzheimer biomarkers. Neurology 89, 1782–1788. 10.1212/wnl.0000000000004569 28939668 PMC5664306

[B11] Cesca F. Baldelli P. Valtorta F. Benfenati F. (2010). The synapsins: key actors of synapse function and plasticity. Prog. Neurobiol. 91, 313–348. 10.1016/j.pneurobio.2010.04.006 20438797

[B12] Choi J. Y. Cui Y. Kim B. G. (2015). Interaction between hypertension and cerebral hypoperfusion in the development of cognitive dysfunction and white matter pathology in rats. Neuroscience 303, 115–125. 10.1016/j.neuroscience.2015.06.056 26143013

[B13] Choi U. B. Strop P. Vrljic M. Chu S. Brunger A. T. Weninger K. R. (2010). Single-molecule FRET-derived model of the synaptotagmin 1-SNARE fusion complex. Nat. Struct. Mol. Biol. 17, 318–324. 10.1038/nsmb.1763 20173763 PMC2922927

[B14] Farkas E. Institóris A. Domoki F. Mihaly A. Luiten P. G. Bari F. (2004). Diazoxide and dimethyl sulphoxide prevent cerebral hypoperfusion-related learning dysfunction and brain damage after carotid artery occlusion. Brain Res. 1008, 252–260. 10.1016/j.brainres.2004.02.037 15145763

[B15] Fossati G. Morini R. Corradini I. Antonucci F. Trepte P. Edry E.. (2015). Reduced SNAP-25 increases PSD-95 mobility and impairs spine morphogenesis. Cell Death Differ. 22, 1425–1436. 10.1038/cdd.2014.227 25678324 PMC4532770

[B16] Frank B. Marcu A. de Oliveira Almeida Petersen A. L. Weber H. Stigloher C. Mottram J. C.. (2015). Autophagic digestion of Leishmania major by host macrophages is associated with differential expression of BNIP3, CTSE and the miRNAs miR-101c, miR-129 and miR-210. Parasit. Vectors 8:404. 10.1186/s13071-015-0974-3 26226952 PMC4521392

[B17] Furuya T. K. Silva P. N. O. Payão S. L. M. Bertolucci P. H. Rasmussen L. T. De Labio R. W.. (2012). Analysis of SNAP25 mRNA expression and promoter DNA methylation in brain areas of Alzheimer’s Disease patients. Neuroscience 220, 41–46. 10.1016/j.neuroscience.2012.06.035 22732502

[B18] Ghanbari M. Erkeland S. J. Xu L. Colijn J. M. Franco O. H. Dehghan A.. (2017). Genetic variants in microRNAs and their binding sites within gene 3’UTRs associate with susceptibility to age-related macular degeneration. Hum. Mutat. 38, 827–838. 10.1002/humu.23226 28397307

[B19] Horváth D. Tamas I. Sipos A. Darula Z. Becsi B. Nagy D.. (2017). Myosin phosphatase and RhoA-activated kinase modulate neurotransmitter release by regulating SNAP-25 of SNARE complex. PLoS One 12:e0177046. 10.1371/journal.pone.0179296 28486561 PMC5423623

[B21] Hu Y.-W. Jiang J.-J. Yan G. Wang R.-Y. Tu G.-J. (2016). MicroRNA-210 promotes sensory axon regeneration of adult mice *in vivo* and *in vitro*. Neurosci. Lett. 622, 61–66. 10.1016/j.neulet.2016.04.034 27102143

[B20] Hu M. Liu Z. Lv P. Wang H. Zhu Y. Qi Q.. (2017). Autophagy and Akt/CREB signalling play an important role in the neuroprotective effect of nimodipine in a rat model of vascular dementia. Behav. Brain Res. 325, 79–86. 10.1016/j.bbr.2016.11.053 27923588

[B22] Imai S. Saeki M. Yanase M. Horiuchi H. Abe M. Narita M.. (2011). Change in microRNAs associated with neuronal adaptive responses in the nucleus accumbens under neuropathic pain. J. Neurosci. 31, 15294–15299. 10.1523/jneurosci.0921-11.2011 22031875 PMC6703523

[B23] Jurado S. Goswami D. Zhang Y. Molina A. J. Sudhof T. C. Malenka R. C. (2013). LTP requires a unique postsynaptic SNARE fusion machinery. Neuron 77, 542–558. 10.1016/j.neuron.2012.11.029 23395379 PMC3569727

[B24] Kasparova S. Brezova V. Valko M. Horecky J. Mlynarik V. Liptaj T.. (2005). Study of the oxidative stress in a rat model of chronic brain hypoperfusion. Neurochem. Int. 46, 601–611. 10.1016/j.neuint.2005.02.006 15863238

[B25] Kube M. Chernikova T. N. Al-Ramahi Y. Beloqui A. Lopez-Cortez N. Guazzaroni M. E.. (2013). SNAP-25 regulates spine formation throgh postsynaptic binding to p140Cap. Nat. Commun. 4:2136. 10.1038/ncomms3136 23868368

[B26] Lee K. Kim J. H. Kwon O. B. An K. Ryu J. Cho K.. (2012). An activity-regulated microRNA, miR-188, controls dendritic plasticity and synaptic transmission by downregulating neuropilin-2. J. Neurosci. 32, 5678–5687. 10.1523/jneurosci.6471-11.2012 22514329 PMC5010781

[B29] Li Y. Chen Z. Gao Y. Pan G. Zheng H. Zhang Y.. (2017a). Synaptic adhesion molecule Pcdh-γC5 mediates synaptic dysfunction in Alzheimer’s disease. J. Neurosci. 37, 9259–9268. 10.1523/jneurosci.1051-17.2017 28842416 PMC5607468

[B28] Li J. Zhang Y. V. Asghari Adib E. Stanchev D. T. Xiong X. Klinedinst S.. (2017b). Restraint of presynaptic protein levels by Wnd/DLK signaling mediates synaptic defects associated with the kinesin-3 motor Unc-104. Elife 6:e24271. 10.7554/elife.24271 28925357 PMC5605197

[B27] Li F. Wei G. Bai Y. Li Y. Huang F. Lin J.. (2015). MicroRNA-574 is involved in cognitive impairment in 5-month-old APP/PS1 mice through regulation of neuritin. Brain Res. 1627, 177–188. 10.1016/j.brainres.2015.09.022 26423933

[B31] Liu Y. Li Y. Ren Z. Si W. Li Y. Wei G.. (2017). MicroRNA-125a-3p is involved in early behavioral disorders in stroke-afflicted rats through the regulation of Cadm2. Int. J. Mol. Med. 40, 1851–1859. 10.3892/ijmm.2017.3179 29039453 PMC5716446

[B30] Liu B. Tang J. Zhang J. Li S. Yuan M. Wang R. (2014). Autophagy activation aggravates neuronal injury in the hippocampus of vascular dementia rats. Neural Regen. Res. 9, 1288–1296. 10.4103/1673-5374.137576 25221581 PMC4160855

[B32] Lou X. Kim J. Hawk B. J. Shin Y. K. (2017). α-Synuclein may cross-bridge v-SNARE and acidic phospholipids to facilitate SNARE-dependent vesicle docking. Biochem. J. 474, 2039–2049. 10.1042/bcj20170200 28495859 PMC5772654

[B33] Ma Q. Dasgupta C. Li Y. Huang L. Zhang L. (2017a). MicroRNA-210 suppresses junction proteins and disrupts blood-brain barrier integrity in neonatal rat hypoxic-ischemic brain injury. Int. J. Mol. Sci. 18:E1356. 10.3390/ijms18071356 28672801 PMC5535849

[B34] Ma X. Xu W. Zhang Z. Liu N. Yang J. Wang M.. (2017b). Salvianolic acid B ameliorates cognitive deficits through IGF-1/Akt pathway in rats with vascular dementia. Cell. Physiol. Biochem. 43, 1381–1391. 10.1159/000481849 28992623

[B35] Magill S. T. Cambronne X. A. Luikart B. W. Lioy D. T. Leighton B. H. Westbrook G. L.. (2010). microRNA-132 regulates dendritic growth and arborization of newborn neurons in the adult hippocampus. Proc. Natl. Acad. Sci. U S A 107, 20382–20387. 10.1073/pnas.1015691107 21059906 PMC2996687

[B36] Miao J. Xu F. Davis J. Otte-Holler I. Verbeek M. M. Van Nostrand W. E. (2005). Cerebral microvascular amyloid β protein deposition induces vascular degeneration and neuroinflammation in transgenic mice expressing human vasculotropic mutant amyloid β precursor protein. Am. J. Pathol. 167, 505–515. 10.1016/s0002-9440(10)62993-8 16049335 PMC1603574

[B37] Miklossy J. (2008). Chronic inflammation and amyloidogenesis in Alzheimer’s disease — role of spirochetes. J. Alzheimers. Dis. 13, 381–391. 10.3233/jad-2008-13401 18487847

[B38] Moravec C. E. Samuel J. Weng W. Wood I. C. Sirotkin H. I. (2016). Maternal Rest/Nrsf regulates zebrafish behavior through snap25a/b. J.Neurosci. 36, 9407–9419. 10.1523/jneurosci.1246-16.2016 27605615 PMC5013188

[B39] Pathania M. Torres-Reveron J. Yan L. Kimura T. Lin T. V. Gordon V.. (2012). miR-132 enhances dendritic morphogenesis, spine density, synaptic integration and survival of newborn olfactory bulb neurons. PLoS One 7:e38174. 10.1371/journal.pone.0038174 22693596 PMC3364964

[B40] Riccardi S. Bergling S. Sigoillot F. Beibel M. Werner A. Leighton-Davies J.. (2016). MiR-210 promotes sensory hair cell formation in the organ of corti. BMC Genomics 17:309. 10.1186/s12864-016-2620-7 27121005 PMC4848794

[B41] Román G. C. Tatemichi T. K. Erkinjuntti T. Cummings J. L. Masdeu J. C. Garcia J. H.. (1993). Vascular dementia: diagnostic criteria for research studies. Neurology 43, 250–260. 10.1212/WNL.43.2.250 8094895

[B42] Scheff S. W. Neltner J. H. Nelson P. T. (2014). Is synaptic loss a unique hallmark of Alzheimer’s disease? Biochem. Pharmacol. 88, 517–528. 10.1016/j.bcp.2013.12.028 24412275 PMC4230706

[B43] Schiavo G. Stenbeck G. Rothman J. E. Söllner T. H. (1997). Binding of the synaptic vesicle v-SNARE, synaptotagmin, to the plasma membrane t-SNARE, SNAP-25, can explain docked vesicles at neurotoxin-treated synapses. Proc. Natl. Acad. Sci. U S A 94, 997–1001. 10.1073/pnas.94.3.997 9023371 PMC19628

[B44] Shi Y. Zhang Y. Lou J. (2017). The influence of cell membrane and SNAP25 linker loop on the dynamics and unzipping of SNARE complex. PLoS One 12:e0176235. 10.1371/journal.pone.0176235 28426820 PMC5398687

[B45] Shu Y. Zhang H. Kang T. Zhang J. J. Yang Y. Liu H.. (2013). PI3K/Akt signal pathway involved in the cognitive impairment caused by chronic cerebral hypoperfusion in rats. PLoS One 8:e81901. 10.1371/journal.pone.0081901 24339978 PMC3858283

[B46] Sørensen J. B. Nagy G. Varoqueaux F. Nehring R. B. Brose N. Wilson M. C.. (2003). Differential control of the releasable vesicle pools by SNAP-25 splice variants and SNAP-23. Cell 114, 75–86. 10.1016/s0092-8674(03)00477-x 12859899

[B47] Sugeno N. Jackel S. Voigt A. Wassouf Z. Schulze-Hentrich J. Kahle P. J. (2016). α-Synuclein enhances histone H3 lysine-9 dimethylation and H3K9me2-dependent transcriptional responses. Sci. Rep. 6:36328. 10.1038/srep36328 27808254 PMC5093762

[B48] van den Maagdenberg A. M. J. M. Plomp J. J. (2003). Neuromuscular synapse function in typical migraine. Cephalalgia 23, 73–74. 10.1046/j.1468-2982.2003.00501.x 12603361

[B49] Van Hook M. J. Babai N. Zurawski Z. Yim Y. Y. Hamm H. E. Thoreson W. B. (2017). A presynaptic group III mGluR recruits Gβγ/SNARE interactions to inhibit synaptic transmission by cone photoreceptors in the vertebrate retina. J. Neurosci. 37, 4618–4634. 10.1523/jneurosci.2948-16.2017 28363980 PMC5413191

[B50] Vrljic M. Strop P. Ernst J. A. Sutton R. B. Chu S. Brunger A. T. (2010). Molecular mechanism of the synaptotagmin-SNARE interaction in Ca2+-triggered vesicle fusion. Nat. Struct. Mol. Biol. 17, 325–331. 10.1038/nsmb.1764 20173762 PMC2928146

[B51] Wan Q. Ma X. Zhang Z. J. Sun T. Xia F. Zhao G.. (2017). Ginsenoside reduces cognitive impairment during chronic cerebral hypoperfusion through brain-derived neurotrophic factor regulated by epigenetic modulation. Mol. Neurobiol. 54, 2889–2900. 10.1007/s12035-016-9868-4 27021024

[B52] Wang J. Zhang S. Ma H. Yang S. Liu Z. Wu X.. (2017). Chronic intermittent hypobaric hypoxia pretreatment ameliorates ischemia-induced cognitive dysfunction through activation of ERK1/2-CREB-BDNF pathway in anesthetized mice. Neurochem. Res. 42, 501–512. 10.1007/s11064-016-2097-4 27822668

[B53] Wang J. Zhang Y. Xu F. (2018a). Function and mechanism of microRNA-210 in acute cerebral infarction. Exp. Ther. Med. 15, 1263–1268. 10.3892/etm.2017.5577 29434712 PMC5774459

[B54] Wang L. Wang F. Liu S. Yang X. Yang J. Ming D. (2018b). VEGF attenuates 2-VO induced cognitive impairment and neuronal injury associated with the activation of PI3K/Akt and Notch1 pathway. Exp. Gerontol. 102, 93–100. 10.1016/j.exger.2017.12.010 29248560

[B55] Wei C. Thatcher E. J. Olena A. F. Cha D. J. Perdigoto A. L. Marshall A. F.. (2013). *miR-153* regulates SNAP-25, synaptic transmission and neuronal development. PLoS One 8:e57080. 10.1371/journal.pone.0057080 23451149 PMC3581580

[B56] Yang Y. Xia Z. Liu Y. (2000). SNAP-25 functional domains in SNARE core complex assembly and glutamate release of cerebellar granule cells. J. Biol. Chem. 275, 29482–29487. 10.1074/jbc.m003237200 10882724

[B57] Yao Y. Wang F. Yang X. Zang D. Yang J. Wang Z. (2018). Bombesin attenuated ischemia-induced spatial cognitive and synaptic plasticity impairment associated with oxidative damage. Biomed. Pharmacother. 103, 87–93. 10.1016/j.biopha.2018.03.155 29635132

[B58] Zampa F. Bicker S. Schratt G. (2018). Activity-Dependent Pre-miR-134 Dendritic Localization Is Required for Hippocampal Neuron Dendritogenesis. Front. Mol. Neurosci. 11:171. 10.3389/fnmol.2018.00171 29942249 PMC6004952

[B59] Zhang H. Y. Zheng C. Y. Yan H. Wang Z. F. Tang L. L. Gao X.. (2008). Potential therapeutic targets of huperzine A for Alzheimer’s disease and vascular dementia. Chem. Biol. Interact. 175, 396–402. 10.1016/j.cbi.2008.04.049 18565502

[B60] Zhao T. Fu Y. Sun H. Liu X. (2018). Ligustrazine suppresses neuron apoptosis via the Bax/Bcl-2 and caspase-3 pathway in PC12 cells and in rats with vascular dementia. IUBMB Life 70, 60–70. 10.1002/iub.1704 29247598

[B61] Zhu W. Wang X.-R. Du S.-Q. Yan C.-Q. Yang N.-N. Lin L.-L.. (2018). Anti-oxidative and Anti-apoptotic Effects of Acupuncture: Role of Thioredoxin-1 in the Hippocampus of Vascular Dementia Rats. Neuroscience 379, 281–291. 10.1016/j.neuroscience.2018.03.029 29592844

[B62] Zurawski Z. Page B. Chicka M. C. Brindley R. L. Wells C. A. Preininger A. M.. (2017). Gβγ directly modulates vesicle fusion by competing with synaptotagmin for binding to neuronal SNARE proteins embedded in membranes. J. Biol. Chem. 292, 12165–12177. 10.1074/jbc.m116.773523 28515322 PMC5519367

