# Supplementary figures and images for: MicroRNA-210-5p Contributes to Cognitive Impairment in Early Vascular Dementia Rat Model Through Targeting Snap25
Source: Front Mol Neurosci. 2018 Nov 13;11:388. doi: 10.3389/fnmol.2018.00388 (PMC6243094; doi:10.3389/fnmol.2018.00388)

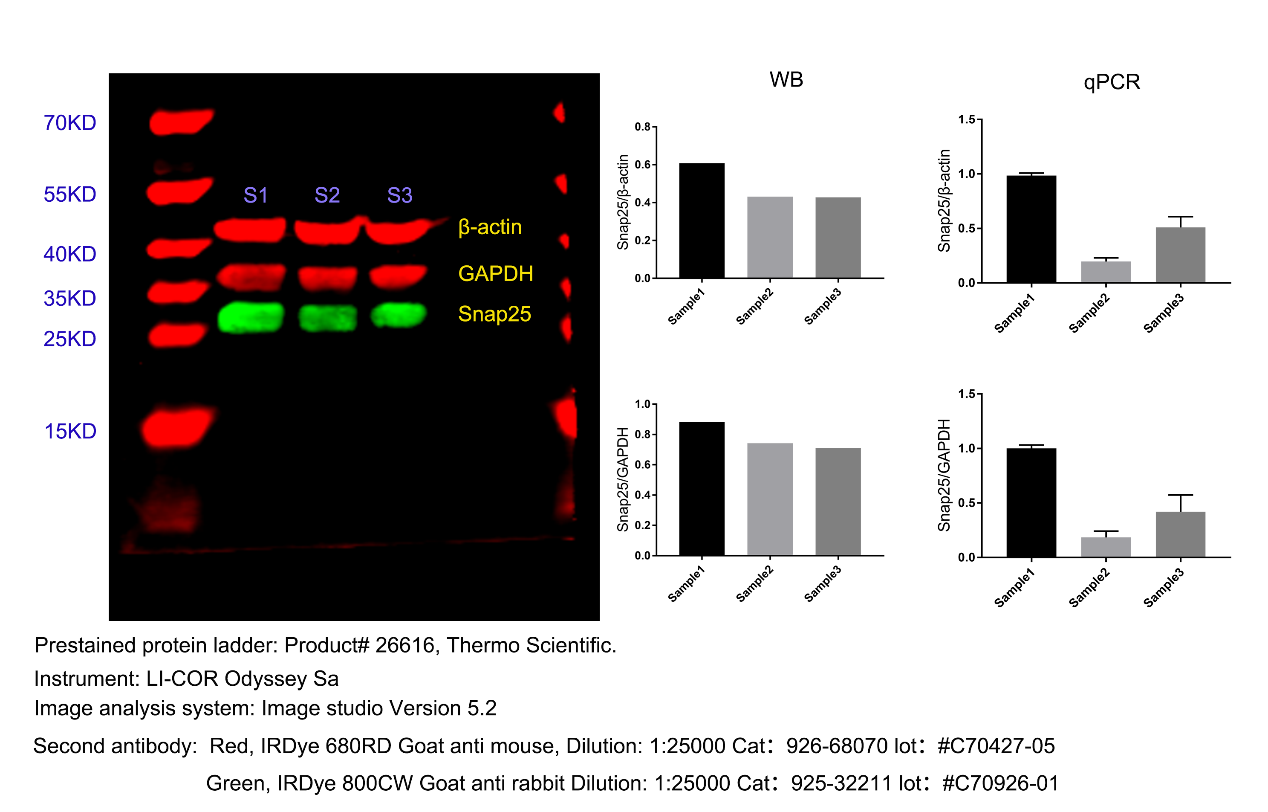

Supplement: Supplementary file 1 [file Data_Sheet_1.docx]
